# DNA screening of *Drosophila suzukii* predators in berry field orchards shows new predatory taxonomical groups

**DOI:** 10.1371/journal.pone.0249673

**Published:** 2021-04-08

**Authors:** Sara Sario, Conceição Santos, Fátima Gonçalves, Laura Torres

**Affiliations:** 1 Faculty of Sciences of University of Porto (FCUP), iB2Lab, Department of Biology, Rua do Campo Alegre, Porto, Portugal; 2 LAQV/REQUIMTE, University of Porto, Porto, Portugal; 3 Centre for the Research and Technology of Agro-Environmental and Biological Sciences (CITAB), University of Trás-os-Montes and Alto Douro (UTAD), Quinta de Prados, Vila Real, Portugal; 4 Centro de Investigação de Montanha (CIMO), Instituto Politécnico de Bragança, Bragança, Portugal; University of Carthage, TUNISIA

## Abstract

*Drosophila suzukii* (spotted wing drosophila, SWD) is a pandemic quarantine pest that attacks mostly red fruits. The high number of life cycles per year, its ability to rapidly invade and spread across new habitats, and highly polyphagous nature, makes this a particularly aggressive invasive species, for which efficient control methods are currently lacking. The use of native natural predators is particularly promising to anchor sustainable and efficient measures to control SWD. While several field studies have suggested the presence of potential predatory species in infested orchards, only a few confirmed the presence of SWD DNA in predators’ gut content. Here, we use a DNA-based approach to identify SWD predators among the arthropod diversity in South Europe, by examining the gut content of potential predator specimens collected in SWD-infested berry fields in North Portugal. These specimens were morphologically identified to the family/order, and their gut content was screened for the presence of SWD DNA using PCR. New SWD predatory taxonomical groups were identified, as Opiliones and Hemerobiidae, in addition to known SWD predators, such as Hemerobiidae, Chrysopidae, Miridae, Carabidae, Formicidae and Araneae. Additionally, the presence of a spider family, Uloboridae, in the orchards was recorded for the first time, posing this family as another SWD-candidate predator. This study sets important bases to further investigate the potential large-scale use of some of these confirmed predator taxa for SWD control in South Europe.

## Introduction

The spotted wing drosophila (SWD), *Drosophila suzukii* (Matsumura), is a pandemic and highly invasive pest that recently arrived to Europe, with first reports in 2010 occurring in Italy and France (data from the European Plant Protection Organization (EPPO) [1]). SWD integrates since 2011 the A2 List of pests recommended for regulation as quarantine pests [2]. Being extremely polyphagous, this pest is able to develop in a wide range of cultivated and wild fruits [3], thus causing severe production losses in different fruit chains of value. Its most susceptible host fruits are thin-skinned berries, including several *Vaccinum* spp. such as cranberries and blueberries, strawberries, or table and wine grapes [4]. But SWD also attacks stone fruits, such as cherries or plums [5,6]. Each SWD female has the capacity to lay hundreds of eggs in its lifespan and a rapid life cycle that easily originates millions of descendants during the months of the fly’s reproductive season (up to 10 generations per year) [7]. In addition, as the larvae feed on the fruit flesh, infested fruits become unmarketable, which associated with loss of crops, pest management and fruit selection, leads to substantial economic losses [8].

Current SWD control methods rely on the use of broad-spectrum insecticides (spinosyns, organophosphates, pyrethroids and neonicotinoids), whose active ingredients work predominantly against adult flies, as the larvae are protected inside the fruits [9]. Other methods such as mass trapping, sanitary measures, netting or the use of kaolin/chalk have also been recommended for SWD control, but they are extremely time consuming and expensive, and end up not improving the control of the pest [10]. Moreover, insecticide resistance has been identified [11], making biological control strategies for SWD attractive approaches that promise to reduce long-term management costs, being also more sustainable and environmentally friendly [12,13].

The success of a biological invader such as SWD depends on reduced impacts of natural enemies in the invaded environments [5,14–19]. Predators and parasitoids are frequently used in biological control and may constitute a relevant strategy to reduce SWD populations, both in wild and crop host species [12,20,21]. A revision of 12 studies on potential predators showed 22 families of interest (excluding spider families, also SWD predators [10,22]), of which only eleven were confirmed as SWD predators, namely Forficulidae (earwigs) [10,23,24], Nabidae (damsel bugs) [10], Formicidae (ants) [25,26], Anthocoridae (pirate bugs) [20,24,25,27,28], Carabidae (ground beetles), Gryllidae (crickets) [29], Chrysopidae (green lacewing, larvae) [20,24] Staphylinidae (rove beetles) [10,30], Mantidae (praying mantis) [22], Miridae (mirid bugs) [20] and Labiduridae (striped earwigs) [28]. Only in three studies the confirmation of predatory activity by SWD DNA presence in the gut content of the predator was shown (S1 Table). Araneae families were addressed in five studies, and 20 families of potential spider predators were reported in literature, of which seven families were confirmed (S2 Table). Of these, only two studies used the presence of SWD DNA in predator’s gut content to confirm predation (S2 Table). Based on the same revision, most of the studies with SWD predators are focused on laboratory assays, while field predation remains relatively understudied. Wolf *et al*. [10] collected candidate SWD predators in Switzerland and detected feeding in earwigs, spiders and predatory bugs; in the United States of America (USA), Schmidt *et al*. [22] also collected field predators, and detected SWD DNA in spiders and in one individual from the Mantidae family. Ballman *et al*. [29], placed SWD pupae on a berry field (USA) in three methods of exposure to predators (fully-exposed, caged and buried) and determined that predation rates were higher in exposed pupae, and although potential predators were collected in the field, the identification of SWD predators was made as laboratory assays. Kamiyama *et al*. [31] detected natural field predation of SWD sentinel pupae in the USA, but specific predator groups were not identified.

Even though previous information about SWD predators can be used to guide local studies, the identification of native field predators is essential to define adequate control measures, not only to avoid the introduction of non-native predators in a region, but also to improve and increase native populations of actual predators, as recommended under a conservation biological control approach. Despite still scarcely used, the confirmation of SWD-DNA in predators’ gut has emerged as the most reliable method to confirm predation [32].

The aim of this study was to identify potential SWD predators among the arthropod diversity in North Portugal, using as case study berry fields, and identify field SWD predation. The results obtained should represent an important first step to the management of the pest by supporting populations of natural enemies present in the region and further contribute to the general knowledge of SWD predators in other environments.

## Materials and methods

### Arthropod collection and identification

**Fig 1:**
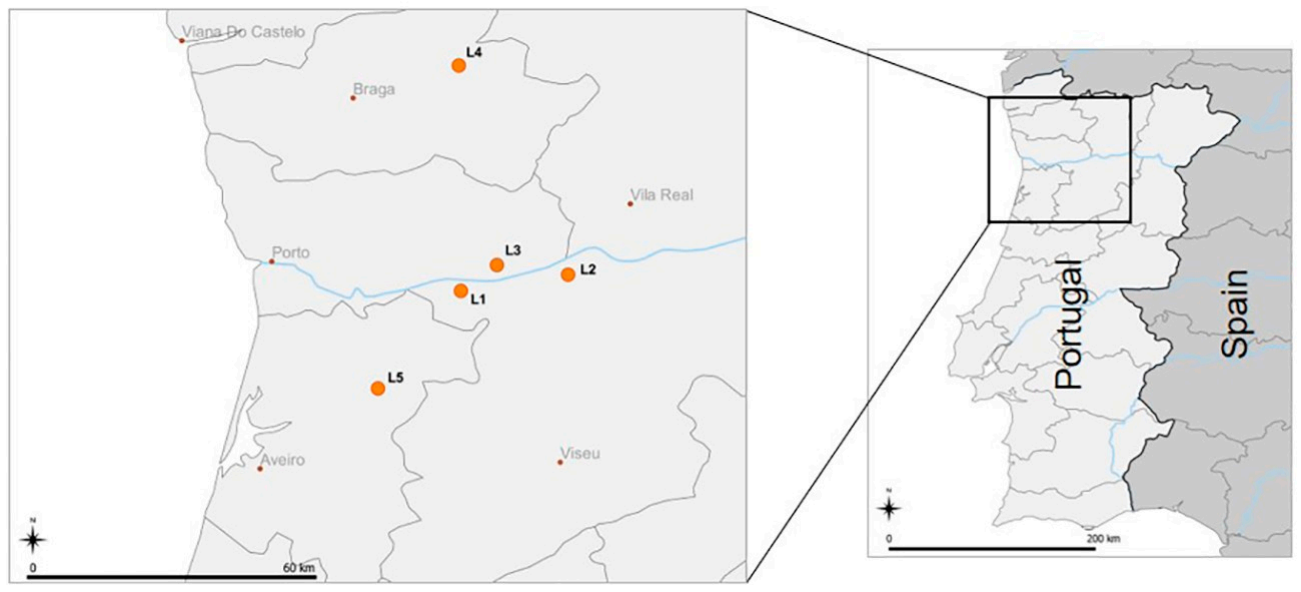
Arthropod sampling locations in Northern Portugal. Scale bar: 60 km (left map), 200 km (right map) Map downloaded from Natural Earth (naturalearthdata.com) and edited in QGIS v3.16.0 (qgis.org).

Arthropods were collected in the Northern region of Portugal, in August 2019, in four blueberry orchards and one blackberry orchard, in five distinct locations: Santiago de Piães (Location 1 (L1); 41.079528,-8.155139; blueberry), São Martinho de Mouros (L2; 41.119639,-7.890639; blueberry), Baião (L3; 41.143306,-8.066306; blueberry), Vieira do Minho (L4; 41.635250,-8.160306; blackberry) and Vale de Cambra (L5; 40.838972,-8.359917; blueberry) (temperature ranged from ≈20–30°C during collection time) (Fig 1). All orchards were developed and maintained under organic farming for 1–5 years upon arthropod collection time; time average size of the orchards was 2 ha, and an average of 6 years of age. All producers used homemade or commercial SWD traps to monitor the pest presence in the orchard; in L2, mass trapping was employed with a *D*. *suzukii* attractive bait. Additionally, the producers used mainly Spinosad as a SWD control agent, following all the legal procedures and recommendations. L1, L3 and L4 had wild vegetation between rows, and only L2 and L5 used mulching films—L2 along the blueberry bush rows, and L5 with the complete orchard’s soil covered. In L4 and L5 nets were used, but while in L4 the nets were only placed above the blackberry trees in order to provide protection against UV-rays, in L5 the net covered the entirety of the orchard as protection against birds. The orchards’ surrounding wild vegetation contained mainly oaks, pine trees, eucalyptus and olive trees; in some cases, there were also vineyards, cherry trees or other fruit trees (L2 and L4). Representative photos of each location are supplied in S1 Fig. The presence of *D*. *suzukii* in the orchard was confirmed by collecting the insect in arthropod samplings and/or in traps used by producers.

Arthropods were collected in three different rows of the orchard: in the first row of bushes in the orchards’ periphery; in the middle of the orchard; and in a row closer to wild vegetation, a water source and/or shade. The wild vegetation present in the last sampled row was characterized by large non-cultivated trees, usually surrounding a small water stream, which contributed to the presence of shade in the nearby rows. On the contrary, the first row in the orchards’ periphery did not have any shade or abundant wild vegetation in the surrounding area.

**Fig 2:**
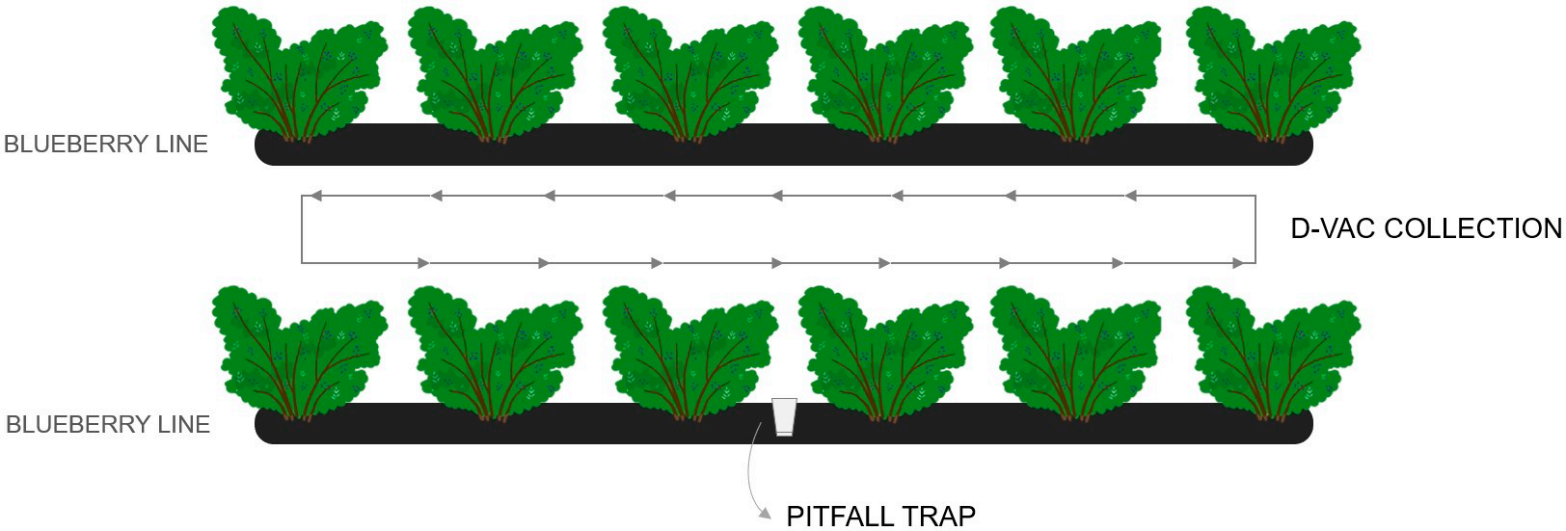
Visual representation of the arthropod sampling methods.

For the arthropod collection, two different methods were used: suction (with a Dietrick Vacuum insect net (D-Vac) machine (Rincon-Vitova Insectaries, Inc., Ventura, CA, US, model 122)), during ≈3min continuously walking along the row (orchards L1-L5), in which arthropods from the canopy were sucked into a meshed bag [17]; and pitfall traps, in which cups with 50% ethanol were inserted on the soil near the shrubs for 24h, for the collection of soil arthropods [33] (L2-L5) (Fig 2). Arthropods collected using D-Vac were etherized on the field by embedding a cotton ball in ether and placing it on a plastic bag with the meshed bag inside during the transportation to the laboratory; they were preserved at 4°C in 15 ml tubes with 96% ethanol until identification. The pitfall contents were transferred to 50 ml tubes and kept at 4°C until arthropod identification.

Collected arthropods were sorted into morphospecies and identified to the order or family level following Triplehorn *et al*. [34]. Moreover, in accordance with the same authors, arthropods were assigned to feeding guilds and grouped accordingly into four trophic categories: detritivores, phytophages, omnivores and predators. Spider family identification was based on Nentwig et al. [35] and specimens were further grouped following Cardoso *et al*. [36]. Thus, families of Araneae were grouped in 7 functional groups based on their foraging strategy (type of web and hunting method), prey range (stenophagous or euryphagous), vertical stratification (ground or foliage) and circadian activity (diurnal or nocturnal): (1) sheet web weavers (ShW), (2) space web weavers (SpW), (3) orb web weavers (OrW), (4) specialists (Sp), (5) ambush hunters (AH), (6) ground hunters (GH), and (7) other hunters (OH). After identification, arthropods belonging to predator and omnivores trophic groups were preserved in 96% ethanol, according to place of collection and family/order, and were kept at 4°C until DNA extraction.

### Screening of field predation

Arthropods identified as potential predators were tested for field predation of *D*. *suzukii*. Potential predators were washed with a 1.5% bleach solution (v/v, in milliQ H_2_O) to remove any traces of *D*. *suzukii* DNA from their body surface. Screening of field predation was assessed by molecular gut-content analysis according to Wolf *et al*. [10]. DNA of potential predators was extracted with GeneMatrix Tissue Purification Kit (EURX Sp.zo.o., Poland) following the manufacturer’s protocol for insect DNA extraction. Arthropods (up to 50mg) were homogenized with PBS (pH = 7,6). The arthropod’s DNA was extracted as a pool within the same family, location and sampling method, except for Araneae families’ DNA, which was extracted according to functional group and sampling method. DNA quality and quantity were analyzed with μDrop^™^ (ThermoFischer Scientific, USA) on a microplate spectrophotometer Multiskan Go (ThermoFischer Scientific, USA). Predation was confirmed by PCR amplification with a primer specific for the identification of *D*. *suzukii* [10]. PCR amplification was employed twice in order to confirm predation vs. no predation results. The reaction was performed with the highest amount of DNA possible for the enzyme (DFS Taq MasterMix, Bioron Life Science, Germany) in order to avoid false negatives due to lack of DNA quantity in the reaction. The reaction was performed in a T100^™^ thermocycler (Bio-Rad Laboratories, USA) and the conditions were 94°C for 2 min, followed by 35 cycles of 94°C for 10 sec, 48°C for 20 sec, 72°C for 15 sec, and a final extension at 72°C for 3 min. A positive control consisting of DNA extracted from a single *D*. *suzukii* was used (previously phenotypically identified according to the EPPO identification key [37]). PCR results were visualized in an 1.4% agarose gel and results were considered positive if a band appeared in the 179bp region.

### Data analysis

The number of arthropods identified as potential SWD predators was expressed as a percentage of the number of all arthropods collected. Differences in species richness and diversity were analysed with ANOVA. For the analysis of row diversity, the total number of potential natural enemies both from D-VAC and pitfall sampling locations was used based on each row location within the orchard. All graphs and statistical analysis were made using GraphPad Prism 8 (GraphPad Software, USA).

## Results

### Arthropod diversity

**Fig 3:**
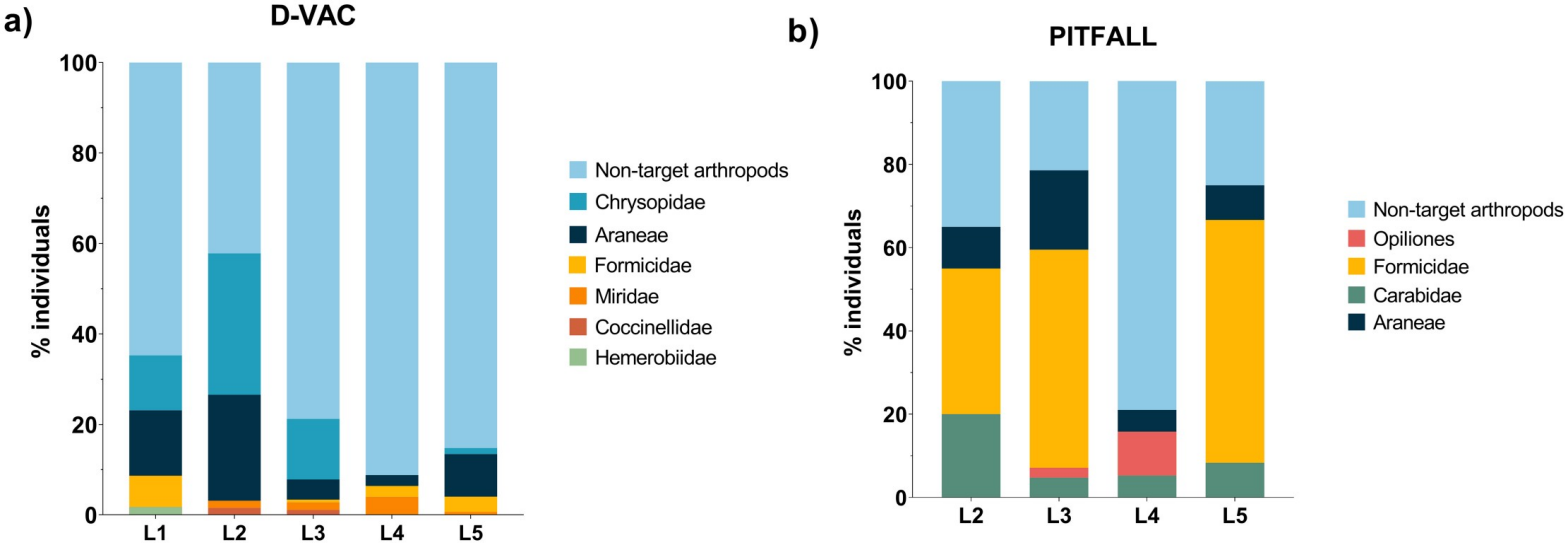
Arthropod diversity collected on berry fields. a) D-Vac collected arthropods; b) Pitfall traps collected arthropods. Values are presented as percentage of individuals from each taxonomical group collected in each location.

Different groups of arthropods were collected in four different blueberry orchards and one blackberry orchard (Table 1). In a total of 169 individuals identified as potential SWD predators collected with D-Vac (representing ≈25% of D-Vac collected arthropods), most belonged to the taxonomic group of Chrysopidae (larva and adults) followed by Araneae, Formicidae, Miridae, Coccinellidae (adults) and Hemerobiidae (adults) (Fig 3a). In the pitfall traps, from a total of 59 arthropods identified as potential predators (≈64% of pitfall collected arthropods), most belonged to Formicidae, followed by Araneae, Carabidae and Opiliones (Fig 3b).

**Table 1:** Number of arthropods collected with D-Vac and pitfall traps.

Trophic/Taxonomic group	D-Vac	Pitfall
***Predators***		
**Arachnida**		
*Araneae*		
Agelenidae	4	1
Araneidae	0	0
Cheiracanthiidae	1	0
Dictynidae	3	0
Lycosidae	0	8
Oxyopidae	35	0
Philodromidae	2	0
Salticidae	3	0
Sparassidae	1	0
Tetragnathidae	0	1
Theridiidae	4	0
Thomisidae	11	0
Uloboridae	1	0
Zodariidae	35	2
*Opiliones*	0	3
**Insecta**		
*Coleoptera*		
Coccinellidae	3	0
Carabidae	0	8
*Heteroptera*		
Miridae	10	0
Other	11	0
*Neuroptera*		
Chrysopidae	67	0
Hemerobiidae	3	0
***Omnivore***		
**Insecta**		
*Hymenoptera*		
Formicidae	21	36
Other	28	1
***Phytophagous***		
**Insecta**		
*Coleoptera*		
Chrysomelidae	11	2
Mordellidae	3	0
Scolytidae	0	1
Tenebrionidae	0	1
Other	0	1
*Hemiptera*		
Cicadomorpha	4	0
Cicadellidae	307	9
Cixiidae	8	0
Membracidae	5	0
Cydnidae	0	2
***Indetermined***		
**Insecta**		
*Diptera*		
Brachycera	115	17
Nematocera	23	0
*Lepidoptera*	5	0

Arthropods are grouped according to their feeding habits and taxonomical group.

**Fig 4:**
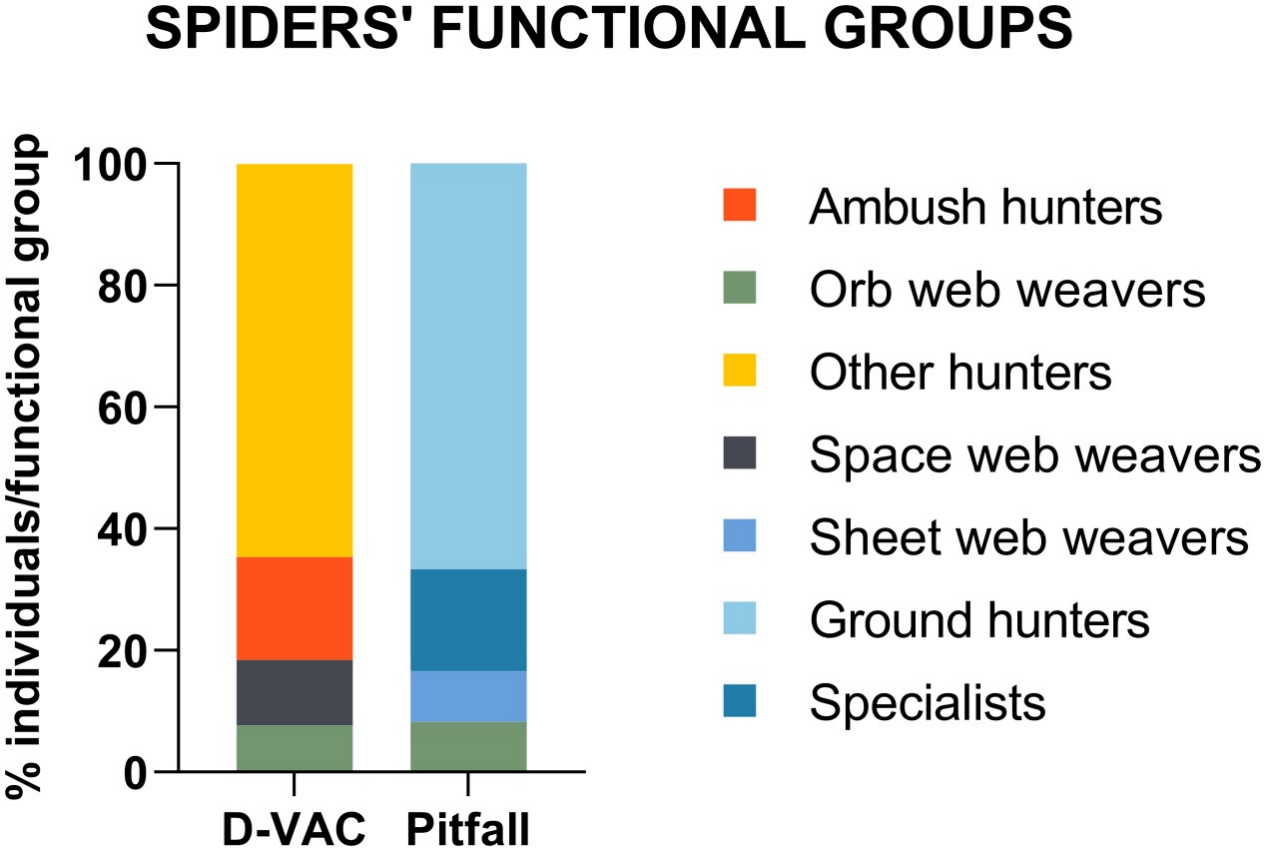
Spiders’ functional group diversity collected with D-Vac and pitfall traps in Portuguese berry fields. Values are presented as percentage of individuals from each functional group collected with D-Vac and Pitfall traps.

**Fig 5:**
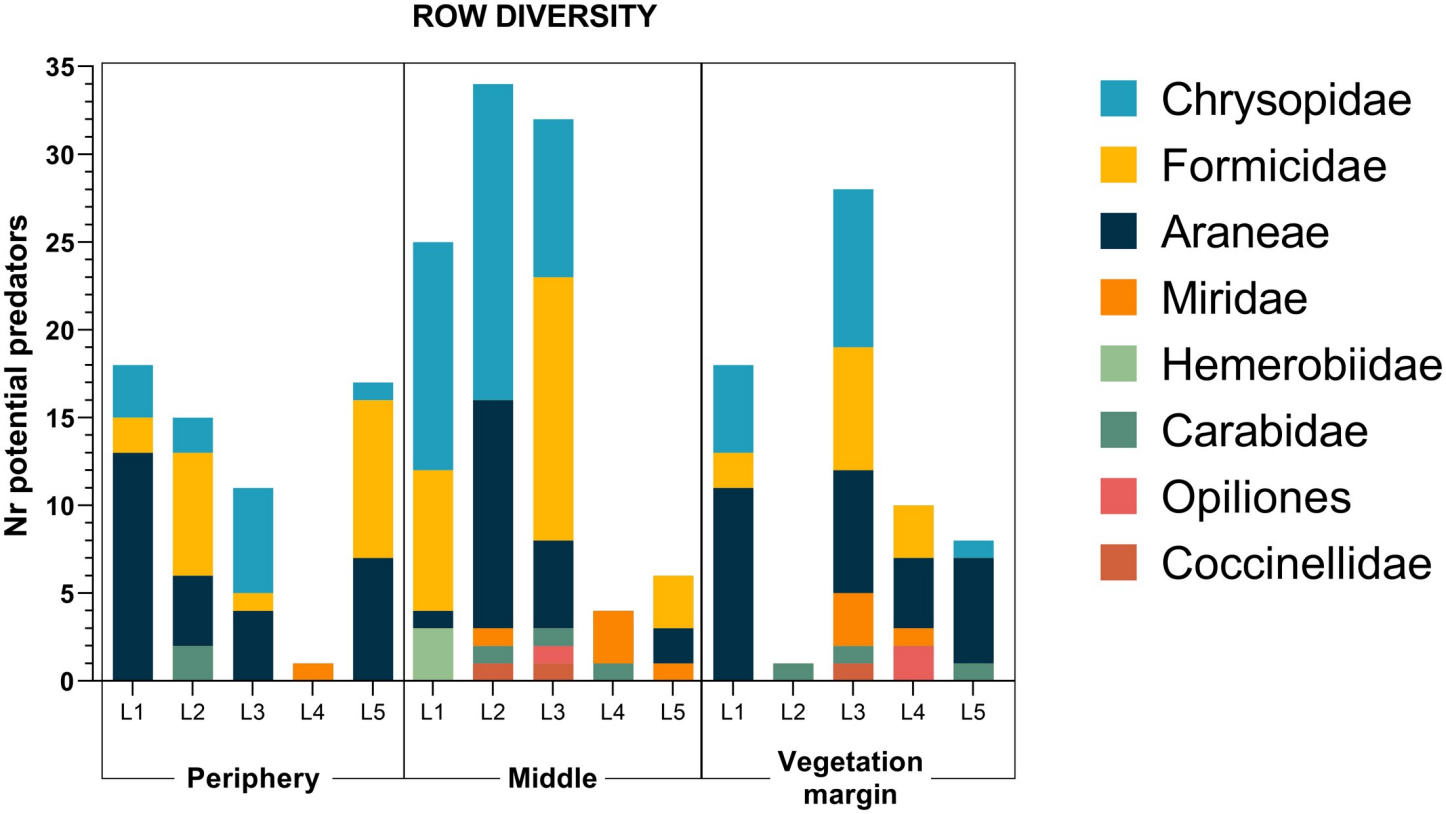
Arthropod row diversity (D-VAC+Pitfall traps). Periphery—row in the periphery of the orchard; Middle—row in the middle of the orchard; Vegetation margin—row closer to wild vegetation, a water source and/or shade. Values are presented as the total number of individuals collected in each row of each location.

We were able to identify 14 different spider families, corresponding to 7 different functional groups (S3 Table), with families related to hunting functional groups being the most abundant in the collections (Fig 4). In all locations, adults of *D*. *suzukii* were collected with D-Vac, confirming the pest presence in the field. The highest number of *D*. *suzukii* was collected on the blackberry fields, with more than 100 individuals (results not shown). When considering the row of the orchard, most potential predators were collected in the middle rows of locations L1, L2 and L3, but in two locations the number was higher near the vegetation margin (L4) and in the periphery (L5) (Fig 5). Although no statistical differences were found between rows concerning species richness and diversity, the highest arthropod diversity was found in the middle row, with arthropods belonging to all of the taxonomic groups identified as potential SWD predators in this study.

### Assessment of field predation

**Fig 6:**
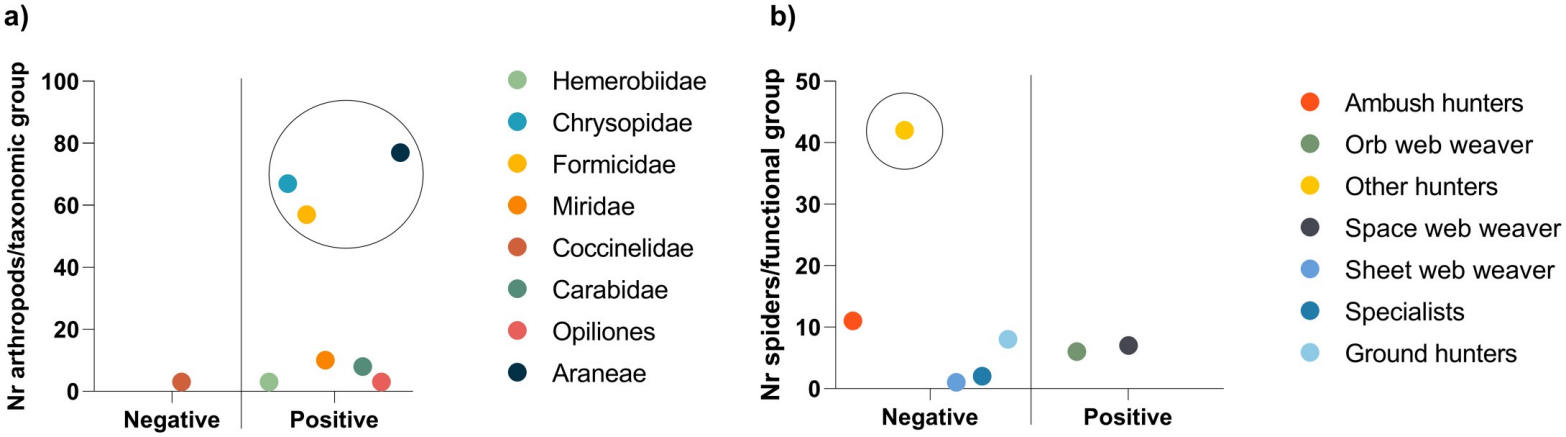
Molecular gut-content analysis of arthropods identified as potential SWD predators. a) PCR amplification results for the 8 families/orders identified, with the representation of the number of identified individuals from each family/order; b) PCR amplification results of the 7 spider functional groups, with the representation of the number of identified individuals from each functional group. Circled are the most relevant taxonomic groups (a) and functional group (b), with the highest number of identified individuals.

**Fig 7:**
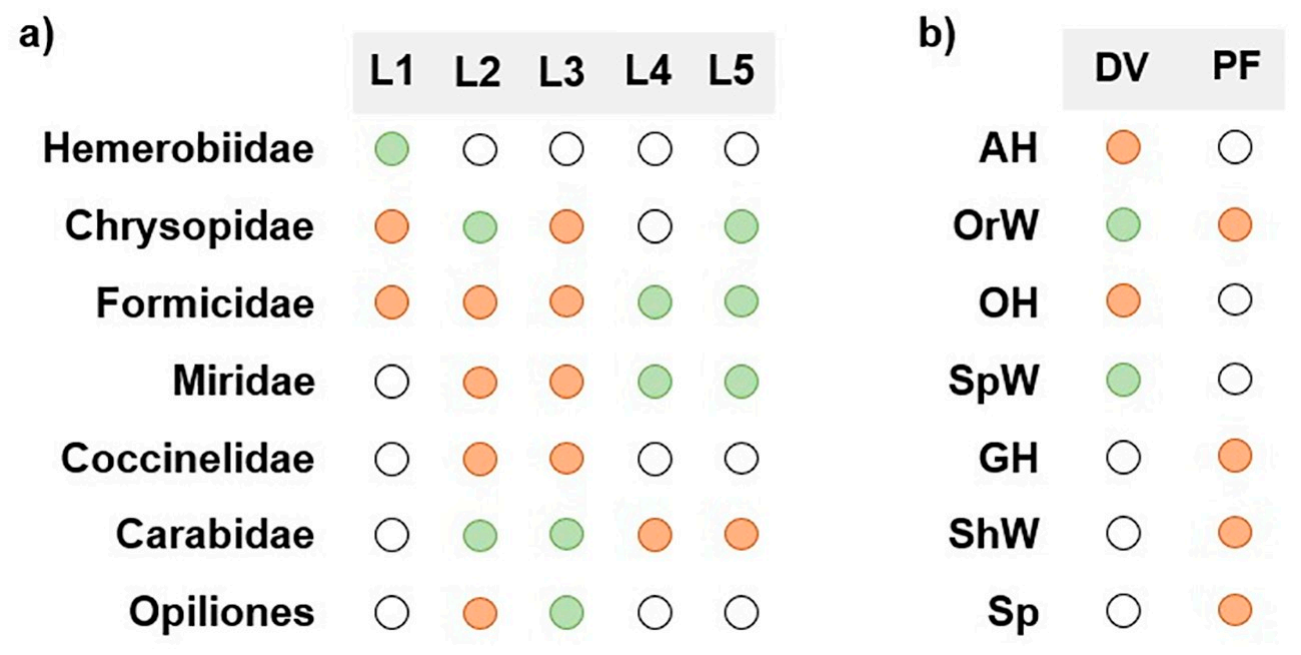
Frequency of *D*. *suzukii* DNA detection in potential predators’ gut-content. a) Detection in 7 of the different familes/orders screened for *D*. *suzukii* DNA presence; b) detection in spiders’ functional groups screened for *D*. *suzukii* DNA presence. Blank dots—predator was not found in the location; orange dots—predator collected in the location but DNA detection was negative; green dots—predator collected in the location with positive DNA detection.

Potential *D*. *suzukii* predators’ gut content was analyzed by PCR amplification of *D*. *suzukii* cytochrome oxidase subunit I (COI) [10]. Of the 8 groups of potential *D*. *suzukii* predators, *D*. *suzukii* DNA was detected in 7, namely Chrysopidae, Formicidae, Miridae, Hemerobiidae, Carabidae, Opiliones and Araneae, with no detection of *D*. *suzukii* DNA in Coccinellidae individuals (Fig 6a). Regarding the Araneae functional groups, *D*. *suzukii* DNA was detected only on 2 of the 7 functional groups, namely orb web weavers (family Araneidae and Uloboridae) and space web weavers (family Dictynidae and Theridiidae), all collected with D-Vac (Fig 6b). The most abundant taxonomic groups with positive results were Chrysopidae, Formicidae and Araneae, however, the most abundant functional group of Araneae, Other hunters (Oxyopidae, Sparassidae, Cheiracanthiidae, Philodromidae and Salticidae), collected with D-Vac, did not predate on *D*. *suzukii*. If frequency of detection is considered, Formicidae was the only group collected in all locations, with positive results in individuals from 3 of the locations (Fig 7a), and both positive results in spiders were from groups collected with D-Vac (Fig 7b).

## Discussion

The need to develop new pest and disease management strategies is increasing. Biological control agents (BCAs) such as bacteria, fungi, parasitoids or predators [12] emerge as the most promising sustainable strategies. Here, we used a DNA-based strategy to produce a detailed catalogue of native predators of SWD in berry fields in Portugal, a critical first step to develop efficient and sustainable control measures.

D-Vac was used to collect predators in plants and surroundings, while pitfall traps collected the potential predators mostly on the ground. Hemerobiidae, Chrysopidae, Miridae and Coccinellidae families were collected in the vegetation, Opiliones and Carabidae on the ground, and Formicidae and Araneae in both cases. Comparing the three sampling rows, predator abundance was higher in the middle of the orchard. Schmidt et al. [22] found a higher abundance of natural enemies in a pine margin of a SWD infested orchard, which correlated with a higher activity of SWD in the same area. The fact that most natural enemies were collected in rows in the middle of the orchard suggests a higher activity of SWD in that area, probably due to higher amount of food.

Of the collected arthropods, only those of the Coccinelidae family were negative for the presence of SWD-DNA specific sequence in the gut. Coccinellids are unspecific predators, and have been referenced as a potential BCA for other insect pests such as e.g., aphids, whiteflies, mealybugs or psyllids (reviewed by Kundoo and Khan [38]). Similar negative results for the presence of SWD in Coccinellids were previously reported in the USA and Switzerland [10,22], supporting coccinellids as unlikely SWD predators.

Families Chrysopidae, Miridae, Carabidae and Formicidae, which field individuals’ gut showed here to contain SWD DNA, had been proposed in laboratory assays as SWD predators. However, laboratorial experiments are often not supported by field data. For example, *Chrysoperla carnea* (Stephens) was in the laboratory a predator of SWD [20,24], but this predation was never confirmed in the field [22,31]. Also, the laboratory predatory activities of the Carabidae *Pterostichus mutus* (Say) and *Bembidion quadrimaculatum* (LeConte) and the Miridae *Dicyphus hesperus* Knight against SWD were not confirmed in the field [13,14]. Field predation of SWD by Formicidae was previously suggested, as ants were observed not only digging and carrying SWD pupae, but also preying on SWD pupae and larvae in dropped blueberries [25,26]. Our data confirm these taxonomic groups as field SWD predators, thus being an important clarification of those previous results.

Despite some inconsistent laboratory vs. field data for SWD predation [13,14,16,23], other studies have shown that Chrysopids are promising predators to be used in the control of other insect pests: *Chrysoperla* spp. larvae might be used to control *Tuta absoluta* (Meyrick) (tomato pinworm) [39], *Frankliniella occidentalis* Pergande (western flower thrips) [40], *Melanaphis sacchari* (Zehntner) (sugarcane aphid) [41] or *Glycaspis brimblecombei* Moore (eucalyptus aphid) [42]. However, the role of chrysopids remains unclear, as the collected individuals were adults, and not all adults of the family Chrysopidae are predators. For example, *Chrysoperla* spp. adults feed on nectar, pollen or honeydew, contrarily to adults of the genus *Chrysopa* that are predators [43].

Miridae, like chrysopsids, are potential BCAs of pests like *T*. *absoluta* [44], *Bactericera cockerelli* (Sulc), *Spodoptera exigua* (Hübner), *Spodoptera frugiperda* (JE Smith) [45,46], all tomato pests; or *Cacopsylla pyri* (Linnaeus), a pear psyllid [47]. In the only study of SWD predation by Miridae species [20], *D*. *hesperus* was confirmed as a SWD predator. Pérez-Hedo *et al*. [48] reviewed the use of mirid bugs as predators in horticultural crops, like tomato. Whilst commercially mirid bugs are already available, such as *D*. *hesperus* (in North America), *Macrolophus pygmeus* (Rambur) and *Nesidiocoris tenuis* (Reuter) (in Europe, Africa and Asia), their use as BCAs in pest management should be further explored [48].

Contrarily to chrysopids and mirid bugs, the use of carabid beetles as BCAs for insect pests has not been suggested. Carabids are generalist predators, feeding on insect pests but also weed seeds, which decreases consumption rates of one or the other [49]. Still, Carabidae species were already reported as predators of pests such as *Ragholetis mendax* Curran (blueberry fruit fly) [50], *Itame argillacearia* Packard (blueberry spanworm), *Altica sylvia* Malloch (blueberry flea beetle) [51] or *Acalymma vittatum* (Fabricius) (striped cucumber beetle) [52].

Among the taxa detected in our work, ants may be those with most potential as a BCA, considering its abundance and capacity to rapidly consume a large number of victims. The use of ant species as a BCA is not recent [53], and besides controlling pests in different agroecosystems [54], they also improve the soil quality [55]. Besides predating on *D*. *suzukii*, ants also predate other pests as *C*. *pyri* [56] and *Hypothenemus hampei* (Ferrari) [57].

This study identified for the first-time arthropods from the Opiliones and Hemerobiidae as SWD predators. Kamiyama *et al*. [31] had recovered Hemerobiidae individuals from traps with sentinel larvae, but did not confirm if the Hemerobiidae individuals were predators or predation was done by individuals from other taxa, such as Formicidae, Anthocoridae or Staphyilinidae [31]. Previous studies showed that Opiliones collected in orchards infested with SWD had negative results for the presence of SWD DNA [10,22]. Hemerobiids are predators of e.g., *Pineus strobi* (Hartig) (pine bark adelgid, a pine tree pest) [58], *Aulacorthum solani* (Kaltenbach) (foxglove aphid, in sweet peppers) [59] or *Planococcus citri* (Risso) (citrus mealybug) [60] but their predatory activity on SWD remains unknown. Similarly, harvestmen (Opiliones) were already identified as predators of *Lobesia botrana* (Denis & Schiffermüller) (grape berry moth) [61] or *A*. *vittatum* (striped cucumber beetle) [52]. Considering our results and that individuals belonging to this taxonomic group were already found in SWD infested orchards in North America and Europe, harvestmen should be considered for further predation studies on SWD, as they may be a suitable predator for the control of this pest.

Here, we also examined the presence of SWD DNA in spiders’ gut content. The use of spiders as BCAs has been explored for a long time now, as they are known as generalist predators, representing a good strategy for certain agroecosystems [62]. Updated insights highlight that the effect of spiders as BCAs depends on the type of pest or existence of alternative preys and environmental conditions (as with any other generalist predator), and on the phenotype of spiders, namely its hunting strategy or behavior (functional group) [63]. Spiders are commonly identified in the field as predators of various pest species, namely *A*. *vittatum* [52], *Frankliniella* spp. [64], or *Phyllocnistis citrella* (Stainton) (citrus leafminer) [65]. In this study, SWD DNA was found in the gut content of spider families belonging to two functional groups, orb web weavers (family Araneidae and Uloboridae) and space web weavers (family Dictynidae and Theridiidae), both web-building groups. Individuals belonging to the family Araneidae and Theridiidae had already been identified as predators of SWD, by testing positive for SWD DNA [10,22], while Dictynidae individuals, although collected in infested orchards, tested negative [22]. Thus, our work is the first report of Uloboridae spiders being collected in SWD infested orchards, although further studies are needed as our study, based on functional groups, did not identify which family specifically predated on SWD, which also applies to the Dictynidae individuals.

When considering the abundance of each predator group in the berry orchards of this study, the most abundant were green lacewings, ants and spiders, suggesting that these are the best candidates for SWD predator-based control, due to their natural abundance in these fields. However, considering the spiders’ functional groups, the most abundant individuals were identified as belonging to the group “other hunters”, which had no positive results for SWD predation. Nonetheless, in previous studies, individuals belonging to this functional group were found positive for SWD-predation [10,22], and it is important to take into consideration that negative results for *D*. *suzukii* DNA presence in predators’ gut may be influenced by a lack of predator-pest encounters at the time of sampling.

Our study did not identify anthocorids as SWD predators even though laboratory assays have focused on this group, namely *Orius* spp. [20,24,27,28]. Only in two instances anthocorids were considered potential SWD predators in field studies, but predation was not confirmed [26,31]. Woltz *et al*. [25] introduced a commercially available anthocorid species in an infested blueberry field with positive predation results, but there was no description if the species was already native to the orchard. Our results illustrated the need to conduct field studies, with the identification of native predators, in order to avoid the introduction of new species exclusively based in laboratory assays.

### Conclusion

This study focused on the assessment of SWD field predation in infested Portuguese berry fields. The results confirmed the presence of SWD predators previously known or suspected, and importantly identified new predatory taxonomical groups, Opiliones and Hemerobiidae. This is the first study to confirm predation of SWD by individuals belonging to these families/orders and to identify the spider family Uloboridae in SWD infested orchards, also with the potential to be a SWD predator. Our results, together with previous studies, encourage additional field studies to further understand the role of the identified predator families/orders as Biological Control Agents, not only of SWD but also other insect pests. Only that will allow determining which native species would be more adequate for pest control, as field predation by these arthropods is still poorly understood.

## Supporting information

S1 Fig Representative photos of each arthropod sampling location. a) Location 1; b) Location 2; c) Location 3; d) Location 4; e) Location 5. L1, L3 and L4 had wild vegetation between rows, and only L2 and L5 used mulching films—L2 along the blueberry shrub rows, and L5 with the complete orchard’s soil covered. In L4 and L5 nets were used, being that in L4 the nets were only placed above the blackberry trees in order to provide protection against UV-rays, and in L5 the net covered the entirety of the orchard as protection against birds.
(PNG)

S1 Table List of potential *D*. *suzukii* predators previously identified. In the **Species** column, when a species is “Not specified”, it indicates that the predator was only identified to the family level. In the **Type of study** column, studies were considered as “Laboratory” when predation trials occurred under controlled conditions in the laboratory; “Field” when the predators or potential predators were captured in the field or predation was observed in the field; when predators were captured in the field but predation trials occurred in the laboratory or both in the laboratory and in the field, studies are identified with “Laboratory” and “Field”. **Predation** was considered “Positive” or “Negative” when specific predators predated or not on *D*. *suzukii*, respectively; it was considered “Not confirmed” when the study identified potential predators and predation was observed, but it was not possible to identify which specific arthropod was the predator. **DNA presence** was considered “Positive” if DNA amplification occurred with SWD specific primers, “Negative” when there was no DNA amplification with specific primers to identify SWD, and “Not tested” when predation was not assessed based on SWD DNA presence. **Origin** refers to the country where the experiments took place, either in the laboratory or field.
(DOCX)

S2 Table List of spiders considered as potential *D*. *suzukii* predators. Spider **families** are grouped according to its **functional group** into Undefined/Specialists, web-building and hunting spiders. Spiders in studies where family was not specified were classified as Spiders. In the **Type of study** column, studies were considered as “Field” when the predators or potential predators were captured in the field or predation was observed in the field. **Predation** was considered “Positive” or “Negative” when specific predators predated or not on *D*. *suzukii*, respectively; it was considered “Not confirmed” when the study identified potential predators and predation was observed, but it was not possible to identify which specific arthropod was the predator. **DNA presence** was considered “Positive” if DNA amplification occurred with SWD specific primers, “Negative” when there was no DNA amplification with specific primers to identify SWD, and “Not tested” when predation was not assessed based on SWD DNA presence. **Origin** refers to the country where the experiments took place, either in the laboratory or field.
(DOCX)

S3 Table Spider families grouped in the corresponding functional group (identified according to Cardoso *et al*. [1]) and general group (web-building spiders, hunting spiders and other spiders). (DOCX)

## References

[1] EPPO. Drosophila suzukii (DROSSU) [World distribution]| EPPO Global Database [Internet]. [cited 2020 Nov 12]. EPPO Reporting Service (2010/007 & 2010/111). https://gd.eppo.int/taxon/DROSSU/distribution.

[2] EPPO. Drosophila suzukii (DROSSU) [Categorization]| EPPO Global Database [Internet]. [cited 2020 Nov 12]. https://gd.eppo.int/taxon/DROSSU/categorization.

[3] Poyet M, Le Roux V, Gibert P, Meirland A, Prévost G, Eslin P, et al. The wide potential trophic niche of the asiatic fruit fly *Drosophila suzukii*: The key of its invasion success in temperate Europe? PLoS One. 2015;10(11). 10.1371/journal.pone.0142785 26581101 PMC4651357

[4] Shrader ME, Burrack HJ, Pfeiffer DG. *Drosophila suzukii* (Diptera: Drosophilidae) oviposition and adult emergence in six wine grape varieties grown in Virginia. J Econ Entomol. 2019;112(1):139–48. 10.1093/jee/toy305 30407506

[5] Asplen MK, Anfora G, Biondi A, Choi DS, Chu D, Daane KM, et al. Invasion biology of spotted wing Drosophila (*Drosophila suzukii*): a global perspective and future priorities. J Pest Sci (2004). 2015;88(3):469–94.

[6] Kenis M, Tonina L, Eschen R, van der Sluis B, Sancassani M, Mori N, et al. Non-crop plants used as hosts by *Drosophila suzukii* in Europe. J Pest Sci (2004). 2016 7 24;89(3):735–48. 10.1007/s10340-016-0755-6 28275324 PMC5318492

[7] Rota-Stabelli O, Blaxter M, Anfora G. Drosophila suzukii. Curr Biol. 2013;23(1):R8–9. 10.1016/j.cub.2012.11.021 23305672

[8] Grumiaux C, Andersen MK, Colinet H, Overgaard J. Fluctuating thermal regime preserves physiological homeostasis and reproductive capacity in *Drosophila suzukii*. J Insect Physiol. 2019 2;113(December 2018):33–41. 10.1016/j.jinsphys.2019.01.001 30615858

[9] Schetelig MF, Lee K-Z, Otto S, Talmann L, Stökl J, Degenkolb T, et al. Environmentally sustainable pest control options for *Drosophila suzukii*. J Appl Entomol. 2018 2;142(1–2):3–17.

[10] Wolf S, Zeisler C, Sint D, Romeis J, Traugott M, Collatz J. A simple and cost-effective molecular method to track predation on *Drosophila suzukii* in the field. J Pest Sci (2004). 2018 3 3;91(2):927–35.

[11] Gress BE, Zalom FG. Identification and risk assessment of spinosad resistance in a California population of *Drosophila suzukii*. Pest Manag Sci. 2019 5 4;75(5):1270–6. 10.1002/ps.5240 30324771

[12] Lee JC, Wang X, Daane KM, Hoelmer KA, Isaacs R, Sial AA, et al. Biological ontrol of spotted-wing Drosophila (Diptera: Drosophilidae)—current and pending tactics. J Integr Pest Manag. 2019;10(1).

[13] Ioriatti C, Anfora G, Grassi A, Puppato S, Rossi Stacconi M V. Current status of the *Drosophila suzukii* control in Europe. In: Acta Horticulturae. International Society for Horticultural Science; 2020. p. 387–96.

[14] Renkema JM, Iglesias LE, Bonneau P, Liburd OE. Trapping system comparisons for and factors affecting populations of *Drosophila suzukii* and *Zaprionus indianus* in winter-grown strawberry. Pest Manag Sci. 2018;74(9):2076–88. 10.1002/ps.4904 29516620

[15] Andreazza F, Bernardi D, dos Santos RSS, Garcia FRM, Oliveira EE, Botton M, et al. *Drosophila suzukii* in Southern Neotropical Region: current tatus and future perspectives. Neotrop Entomol. 2017 12 30;46(6):591–605. 10.1007/s13744-017-0554-7 28852987

[16] Chabert S, Allemand R, Poyet M, Eslin P, Gibert P. Ability of European parasitoids (Hymenoptera) to control a new invasive Asiatic pest, *Drosophila suzukii*. Biol Control. 2012 10 1;63(1):40–7.

[17] Gonçalves MF, Santos SAP, Torres LM. Efficacy of spinosad bait sprays to control *Bactrocera oleae* and impact on non-target arthropods. Phytoparasitica. 2012 2 29;40(1):17–28.

[18] Rossi Stacconi MV, Amiresmaeili N, Biondi A, Carli C, Caruso S, Dindo ML, et al. Host location and dispersal ability of the cosmopolitan parasitoid *Trichopria drosophilae* released to control the invasive spotted wing Drosophila. Biol Control. 2018 2 1;117:188–96.

[19] Mazzetto F, Marchetti E, Amiresmaeili N, Sacco D, Francati S, Jucker C, et al. Drosophila parasitoids in northern Italy and their potential to attack the exotic pest *Drosophila suzukii*. J Pest Sci (2004). 2016 7 1;89(3):837–50.

[20] Bonneau P, Renkema J, Fournier V, Firlej A. Ability of *Muscidifurax raptorellus* and other parasitoids and predators to control *Drosophila suzukii* populations in raspberries in the laboratory. Insects. 2019;10(3). 10.3390/insects10030068 30866498 PMC6468634

[21] Wang X, Lee JC, Daane KM, Buffington ML, Hoelmer KA. Biological control of *Drosophila suzukii*. CAB Rev Perspect Agric Vet Sci Nutr Nat Resour. 2020 12 1;15(054). 10.1093/jee/toz265 31589742

[22] Schmidt JM, Whitehouse TS, Green K, Krehenwinkel H, Schmidt-Jeffris R, Sial AA. Local and landscape-scale heterogeneity shape spotted wing drosophila (*Drosophila suzukii*) activity and natural enemy abundance: Implications for trophic interactions. Agric Ecosyst Environ. 2019;272(November 2018):86–94.

[23] Bourne A, Fountain MT, Wijnen H, Shaw B. Potential of the European earwig (Forficula auricularia) as a biocontrol agent of the soft and stone fruit pest *Drosophila suzukii*. Pest Manag Sci. 2019;75(12):3340–5. 10.1002/ps.5459 31066201

[24] Englert C, Herz A. Acceptability of *Drosophila suzukii* as prey for common predators occurring in cherries and berries. J Appl Entomol. 2019;143(4):387–96.

[25] Woltz JM, Donahue KM, Bruck DJ, Lee JC. Efficacy of commercially available predators, nematodes and fungal entomopathogens for augmentative control of *Drosophila suzukii*. J Appl Entomol. 2015;139(10):759–70.

[26] Woltz JM, Lee JC. Pupation behavior and larval and pupal biocontrol of *Drosophila suzukii* in the field. Biol Control. 2017 7;110(April):62–9.

[27] Cuthbertson AGS, Blackburn LF, Audsley N. Efficacy of commercially available invertebrate predators against *Drosophila suzukii*. Insects. 2014;5(4):952–60. 10.3390/insects5040952 26462951 PMC4592616

[28] Gabarra R, Riudavets J, Rodríguez GA, Pujade-Villar J, Arnó J. Prospects for the biological control of *Drosophila suzukii*. BioControl. 2015;60(3):331–9.

[29] Ballman ES, Collins JA, Drummond FA. Pupation behavior and predation on *Drosophila suzukii* (Diptera: Drosophilidae) pupae in Maine wild blueberry fields. J Econ Entomol. 2017;110(6):2308–17. 10.1093/jee/tox233 29029219

[30] Renkema JM, Telfer Z, Gariepy T, Hallett RH. *Dalotia coriaria* as a predator of *Drosophila suzukii*: Functional responses, reduced fruit infestation and molecular diagnostics. Biol Control. 2015;89:1–10.

[31] Kamiyama MT, Schreiner Z, Guédot C. Diversity and abundance of natural enemies of *Drosophila suzuki*i in Wisconsin, USA fruit farms. BioControl. 2019;64(6):665–76.

[32] González-Chang M, Wratten SD, Lefort MC, Boyer S. Food webs and biological control: A review of molecular tools used to reveal trophic interactions in agricultural systems. Vol. 9, Food Webs. Elsevier Inc.; 2016. p. 4–11.

[33] Moreau CS, Wray BD, Czekanski-Moir JE, Rubin BER. DNA preservation: a test of commonly used preservatives for insects. Invertebr Syst. 2013 3 13;27(1):81.

[34] Triplehorn C, Jhonson N. Borror and DeLong’s Introduction to the Study of Insects. 7th Editio. Thomson Brooks/Cole; 2005.

[35] Nentwig W, Blick T, Gloor D, Hänggi A, Kropf C. Araneae—spiders of Europe. Nentwig W, Blick T, Gloor D, Hänggi A, Kropf C, editors.

[36] Cardoso P, Pekár S, Jocqué R, Coddington JA. Global patterns of guild composition and functional diversity of spiders. PLoS One. 2011;6(6). 10.1371/journal.pone.0021710 21738772 PMC3126856

[37] OEPP/EPPO. PM 7/115 (1) *Drosophila suzukii*. EPPO Bull. 2013 12;43(3):417–24.

[38] Kundoo AA, Khan AA. Coccinellids as biological control agents of soft bodied insects: A review. J Entomol Zool Stud. 2017;5(5 R):1362–73.

[39] Ismoilov K, Wang M, Jalilov A, Zhang X, Lu Z, Saidov A, et al. First report using a native lacewing species to control *Tuta absoluta*: From laboratory trials to field assessment. Insects. 2020;11(5):1–12. 10.3390/insects11050286 32392851 PMC7290810

[40] Luna-Espino HM, Jiménez-Pérez A, Castrejón-Gómez VR. Assessment of *Chrysoperla comanche* (Banks) and *Chrysoperla externa* (Hagen) as biological control agents of *Frankliniella occidentalis* (Pergande) (Thysanoptera: Thripidae) on tomato (*Solanum lycopersicum*) under glasshouse conditions. Insects. 2020 1 29;11(2):87. 10.3390/insects11020087 PMC7073685 32013231

[41] Delgado-Ramírez CS, Salas-Araiza MD, Martínez-Jaime OA, Guzmán-Mendoza R, Flores-Mejia S. Predation capability of *Hippodamia convergens* (Coleoptera: Coccinellidae) and *Chrysoperla carnea* (Neuroptera: Chrysopidae) feeding of *Melanaphis sacchari* (Hemiptera: Aphididae). Florida Entomol. 2019;102(1):24–8.

[42] Cuello EM, Andorno A V., Hernández CM, López SN. Prey consumption and development of the indigenous lacewing *Chrysoperla externa* feeding on two exotic *Eucalyptus* pests. Biocontrol Sci Technol. 2019 12 2;29(12):1159–71.

[43] Serée L, Rouzes R, Thiéry D, Rusch A. Temporal variation of the effects of landscape composition on lacewings (Chrysopidae: Neuroptera) in vineyards. Agric For Entomol. 2020 8 6;22(3):274–83.

[44] Pérez-Aguilar DA, Soares MA, Passos LC, Martínez AM, Pineda S, Carvalho GA. Lethal and sublethal effects of insecticides on *Engytatus varians* (Heteroptera: Miridae), a predator of *Tuta absoluta* (Lepidoptera: Gelechiidae). Ecotoxicology. 2018 8 1;27(6):719–28. 10.1007/s10646-018-1954-0 29923076

[45] Pineda S, Hernández-Quintero O, Velázquez-Rodríguez YB, Viñuela E, Figueroa JI, Morales SI, et al. Predation by *Engytatus varians* (Distant) (Hemiptera: Miridae) on *Bactericera cockerelli* (Sulcer) (Hemiptera: Triozidae) and two Spodoptera species. Bull Entomol Res. 2020 4 1;110(2):270–7. 10.1017/S0007485319000579 31495348

[46] Pérez-Aguilar DA, Martínez AM, Viñuela E, Figueroa JI, Gómez B, Morales SI, et al. Impact of the zoophytophagous predator *Engytatus varians* (Hemiptera: Miridae) on *Bactericera cockerelli* (Hemiptera: Triozidae) control. Biol Control. 2019 5 1;132:29–35.

[47] Ramírez‐Soria MJ, Wäckers F, Sanchez JA. When natural enemies go to sleep: diapause induction and termination in the pear psyllid predator *Pilophorus gallicus* (Hemiptera: Miridae). Pest Manag Sci. 2019 12 15;75(12):3293–301. 10.1002/ps.5451 31006973

[48] Pérez‐Hedo M, Riahi C, Urbaneja A. Use of zoophytophagous mirid bugs in horticultural crops: current challenges and future perspectives. Pest Manag Sci. 2020 9 11;ps.6043. 10.1002/ps.6043 32776672

[49] De Heij SE, Willenborg CJ. Connected carabids: network interactions and their impact on biocontrol by carabid beetles. Bioscience. 2020 6 1;70(6):490–500. 10.1093/biosci/biaa039 32536691 PMC7277018

[50] Renkema JM, Lynch DH, Cutler GC, MacKenzie K, Walde SJ. Predation by *Pterostichus melanarius* (Illiger) (Coleoptera: Carabidae) on immature *Rhagoletis mendax* Curran (Diptera: Tephritidae) in semi-field and field conditions. Biol Control. 2012 1 1;60(1):46–53.

[51] Renkema JM, Christopher Cutler G, Rutherford K. Molecular analysis reveals lowbush blueberry pest predation rates depend on ground beetle (Coleoptera: Carabidae) species and pest density. BioControl. 2014 8 29;59(6):749–60.

[52] Mabin MD, Welty C, Gardiner MM. Predator richness predicts pest suppression within organic and conventional summer squash (*Cucurbita pepo* L. Cucurbitales: Cucurbitaceae). Agric Ecosyst Environ. 2020 1 1;287:106689.

[53] Van Mele P. A historical review of research on the weaver ant Oecophylla in biological control. Agric For Entomol. 2007 11 1;0(0):071101073646001-???.

[54] Frizzo TLM, Souza LM, Sujii ER, Togni PHB. Ants provide biological control on tropical organic farms influenced by local and landscape factors. Biol Control. 2020 12 1;151:104378.

[55] Drummond F, Choate B. Ants as biological control agents in agricultural cropping systems. Terr Arthropod Rev. 2011 6 28;4(2):157–80.

[56] Sanchez JA, Carrasco‐Ortiz A, López‐Gallego E, La Spina M. Ants (Hymenoptera: Formicidae) reduce the density of *Cacopsylla pyri* (Linnaeus, 1761) in Mediterranean pear orchards. Myrmecological News. 2020;30:93–102.

[57] Morris JR, Jiménez-Soto E, Philpott SM, Perfecto I. Ant-mediated (Hymenoptera: Formicidae) biological control of the coffee berry borer: Diversity, ecological complexity, and conservation biocontrol. Myrmecological News. 2018 2 1;26:1–17.

[58] Wantuch HA, Havill NP, Hoebeke ER, Kuhar TP, Salom SM. Predators associated with the pine bark adelgid (hemiptera: Adelgidae), a native insect in appalachian forests, United States of America, in its southern range. Can Entomol. 2019 2 1;151(1):73–84.

[59] Rocca M, Messelink GJ. Combining lacewings and parasitoids for biological control of foxglove aphids in sweet pepper. J Appl Entomol. 2017 6 1;141(5):402–10.

[60] Yayla M, Tusun A, Satar S. The potential of *Sympherobius pygmaeus* (Rambur, 1842) as a biological agent against *Planococcus citri* (Risso, 1813) in citrus orchards. J Entomol Res Soc. 2020;22(1):1–12.

[61] Papura D, Roux P, Joubard B, Razafimbola L, Fabreguettes O, Delbac L, et al. Predation of grape berry moths by harvestmen depends on landscape composition. Biol Control. 2020 11 1;150:104358.

[62] Riechert SE, Lockley T. Spiders as Biological Control Agents. Annu Rev Entomol. 1984 1 28;29(1):299–320.

[63] Michalko R, Pekár S, Entling MH. An updated perspective on spiders as generalist predators in biological control. Vol. 189, Oecologia. Springer Verlag; 2019. p. 21–36. 10.1007/s00442-018-4313-1 30535723

[64] Rocha FH, Infante F, Castillo A, Ibarra-Nuñez G, Goldarazena A, Funderburk JE, et al. Natural enemies of the *Frankliniella* complex species (Thysanoptera: Thripidae) in Ataulfo Mango agroecosystems. J Insect Sci. 2015 1 1;15(1):114. 10.1093/jisesa/iev096 26246440 PMC4675721

[65] Khfif K, Baala M, Walters SA, Bouharroud R, Sbaghi M. Chemical and biological approaches for citrus leafminer *Phyllocnistis citrella* stainton control in a Clementine Orchard, in Moulouya region of Morocco. Arch Phytopathol Plant Prot. 2020 9 13;53(15–16):749–64.

