# Supplementary figures and images for: DNA screening of Drosophila suzukii predators in berry field orchards shows new predatory taxonomical groups
Source: PLoS One. 2021 Apr 8;16(4):e0249673. doi: 10.1371/journal.pone.0249673 (PMC8031375; doi:10.1371/journal.pone.0249673)

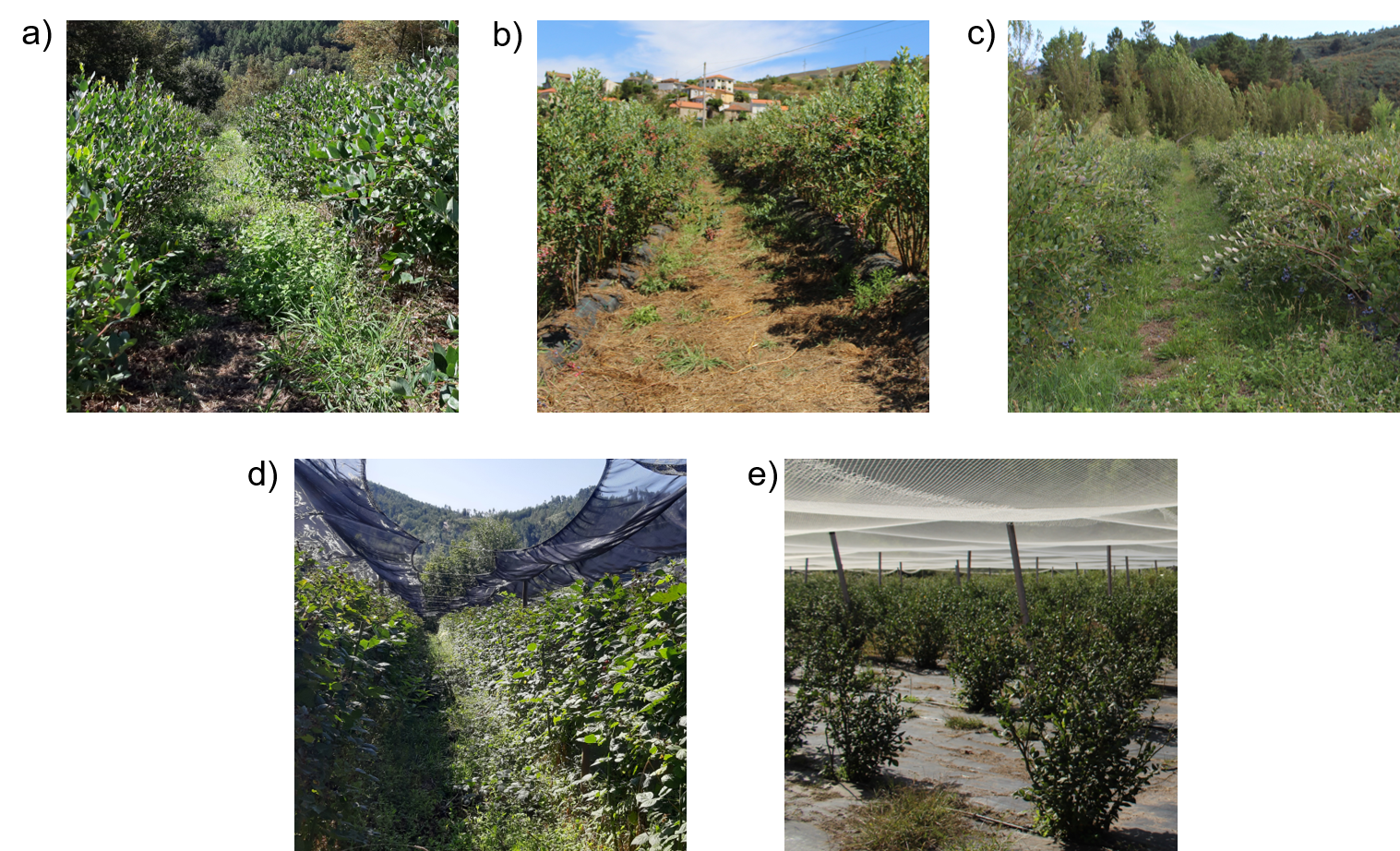

Supplement: S1 Fig — a) Location 1; b) Location 2; c) Location 3; d) Location 4; e) Location 5. L1, L3 and L4 had wild vegetation between rows, and only L2 and L5 used mulching films—L2 along the blueberry shrub rows, and L5 with the complete orchard’s soil covered. In L4 and L5 nets were used, being that in L4 the nets were only placed above the blackberry trees in order to provide protection against UV-rays, and in L5 the net covered the entirety of the orchard as protection against birds. (PNG) [file pone.0249673.s001.png]
