# Supplementary material for: DNA screening of Drosophila suzukii predators in berry field orchards shows new predatory taxonomical groups
Source: PLoS One. 2021 Apr 8;16(4):e0249673. doi: 10.1371/journal.pone.0249673 (PMC8031375; doi:10.1371/journal.pone.0249673)
Supplement: S1 Table — In the Species column, when a species is “Not specified”, it indicates that the predator was only identified to the family level. In the Type of study column, studies were considered as “Laboratory” when predation trials occurred under controlled conditions in the laboratory; “Field” when the predators or potential predators were captured in the field or predation was observed in the field; when predators were captured in the field but predation trials occurred in the laboratory or both in the laboratory and in the field, studies are identified with “Laboratory” and “Field”. Predation was considered “Positive” or “Negative” when specific predators predated or not on D. suzukii, respectively; it was considered “Not confirmed” when the study identified potential predators and predation was observed, but it was not possible to identify which specific arthropod was the predator. DNA presence was considered “Positive” if DNA amplification occurred with SWD specific primers, “Negative” when there was no DNA amplification with specific primers to identify SWD, and “Not tested” when predation was not assessed based on SWD DNA presence. Origin refers to the country where the experiments took place, either in the laboratory or field. (DOCX) [file pone.0249673.s002.docx]

**Table S1.** List of potential *D. suzukii* predators previously identified. In the **Species** column, when a species is “Not specified”, it indicates that the predator was only identified to the family level. In the **Type of study** column, studies were considered as “Laboratory” when predation trials occurred under controlled conditions in the laboratory; “Field” when the predators or potential predators were captured in the field or predation was observed in the field; when predators were captured in the field but predation trials occurred in the laboratory or both in the laboratory and in the field, studies are identified with “Laboratory” and “Field”. **Predation** was considered “Positive” or “Negative” when specific predators predated or not on *D. suzukii*, respectively; it was considered “Not confirmed” when the study identified potential predators and predation was observed, but it was not possible to identify which specific arthropod was the predator. **DNA presence** was considered “Positive” if DNA amplification occurred with SWD specific primers, “Negative” when there was no DNA amplification with specific primers to identify SWD, and “Not tested” when predation was not assessed based on SWD DNA presence. **Origin** refers to the country where the experiments took place, either in the laboratory or field.

| Family | Species | Type of study | Predation | DNA presence | Origin | Reference |
| --- | --- | --- | --- | --- | --- | --- |
| Anthocoridae | *Orius insidiosus* | Laboratory, Field | Positive | Not tested | Canada  USA | [1,2] |
|  | *Orius majusculus* | Laboratory | Positive | Not tested | UK  Germany | [3,4] |
|  | *Orius laevigatus* | Laboratory | Positive | Not tested | UK  Spain | [3,5] |
|  | *Anthocoris nemoralis* | Laboratory | Positive | Not tested | UK | [3] |
|  | Not specified | Field | Not confirmed | Not tested | USA | [6,7] |
| Cantharidae | Not specified | Field | Not confirmed | Not tested | USA | [6] |
| Carabidae | *Pterostichus mutus* | Laboratory | Positive | Not tested | USA | [8] |
|  | *Bembidion quadrimaculatum* | Laboratory | Positive | Not tested | USA | [8] |
|  | Not specified | Field | Negative | Negative | USA | [9] |
| Chrysopidae | *Chrysoperla carnea* | Laboratory | Positive | Not tested | Canada  Germany | [1,4] |
|  | Not specified | Field | Negative | Negative | USA | [9] |
|  |  |  | Not confirmed | Not tested | USA | [7] |
| Coccinellidae | Not specified | Field | Negative | Negative | USA  Switzerland | [9,10] |
|  |  |  | Not confirmed | Not tested | USA | [7] |
| Coenagrionidae | Not specified | Field | Negative | Negative | USA | [9] |
| Coniopterygidae | Not specified | Field | Not confirmed | Not tested | USA | [7] |
| Forficulidae | *Forficula auricularia* | Field | Positive | Positive | Switzerland | [10] |
|  |  | Field, Laboratory | Positive | Not tested | UK  Germany | [4,11] |
|  | Not specified | Field | Negative | Negative | USA | [9] |
|  |  |  | Not confirmed | Not tested | USA | [6] |
| Formicidae | Not specified | Field | Not confirmed | Not tested | USA | [7,8] |
|  |  |  | Positive | Not tested | USA | [2,6] |
| Geocoridae | Not specified | Field | Negative | Negative | USA | [9] |
| Gryllidae | *Gryllus pennsylvanicus* | Laboratory | Positive | Not tested | USA | [8] |
| Hemerobiidae | Not specified | Field | Not confirmed | Not tested | USA | [7] |
| Labiduridae | *Labidura riparia* | Laboratory | Positive | Not tested | Spain | [5] |
| Laelapidae | *Hypoaspis miles* | Laboratory | Negative | Not tested | UK | [3] |
| Mantidae | Not specified | Field | Positive | Positive | USA | [9] |
| Miridae | *Dicyphus hesperus* | Laboratory | Positive | Not tested | Canada | [1] |
| Nabidae | *Himacerus mirmicoides* | Laboratory | Positive | Positive | Germany | [10] |
|  | Not specified | Field | Not confirmed | Not tested | USA | [6] |
| Opiliones | Not specified | Field | Negative | Negative | USA  Switzerland | [9,10] |
|  |  |  | Not confirmed | Not tested | USA | [6,8] |
| Pentatomidae | *Podisus maculiventris* | Laboratory | Negative | Not tested | Canada | [1] |
| Reduviidae | Not specified | Field | Negative | Negative | USA | [9] |
|  |  |  | Not confirmed | Not tested | USA | [6] |
| Staphylinidae | *Atheta coriaria* | Laboratory | Negative | Not tested | UK | [3] |
|  | *Dalotia coriaria* | Laboratory | Positive | Positive | USA | [12] |
|  |  | Laboratory, Field | Negative | Not tested | USA | [2] |
|  | Not specified | Field | Negative | Negative | USA | [9] |
|  |  |  | Positive | Positive | Switzerland | [10] |
|  |  |  | Not confirmed | Not tested | USA | [6,7] |
| Syrphidae | Not specified | Field | Negative | Negative | USA | [9] |

**References**

1. Bonneau P, Renkema J, Fournier V, Firlej A. Ability of muscidifurax raptorellus and other parasitoids and predators to control drosophila suzukii populations in raspberries in the laboratory. Insects. 2019;10(3).

2. Woltz JM, Donahue KM, Bruck DJ, Lee JC. Efficacy of commercially available predators, nematodes and fungal entomopathogens for augmentative control of Drosophila suzukii. J Appl Entomol. 2015;139(10):759–70.

3. Cuthbertson AGS, Blackburn LF, Audsley N. Efficacy of commercially available invertebrate predators against Drosophila suzukii. Insects. 2014;5(4):952–60.

4. Englert C, Herz A. Acceptability of Drosophila suzukii as prey for common predators occurring in cherries and berries. J Appl Entomol. 2019;143(4):387–96.

5. Gabarra R, Riudavets J, Rodríguez GA, Pujade-Villar J, Arnó J. Prospects for the biological control of Drosophila suzukii. BioControl. 2015;60(3):331–9.

6. Woltz JM, Lee JC. Pupation behavior and larval and pupal biocontrol of Drosophila suzukii in the field. Biol Control. 2017 Jul;110(April):62–9.

7. Kamiyama MT, Schreiner Z, Guédot C. Diversity and abundance of natural enemies of Drosophila suzukii in Wisconsin, USA fruit farms. BioControl. 2019;64(6):665–76.

8. Ballman ES, Collins JA, Drummond FA. Pupation Behavior and Predation on Drosophila suzukii (Diptera: Drosophilidae) Pupae in Maine Wild Blueberry Fields. J Econ Entomol. 2017;110(6):2308–17.

9. Schmidt JM, Whitehouse TS, Green K, Krehenwinkel H, Schmidt-Jeffris R, Sial AA. Local and landscape-scale heterogeneity shape spotted wing drosophila (Drosophila suzukii) activity and natural enemy abundance: Implications for trophic interactions. Agric Ecosyst Environ. 2019;272(November 2018):86–94.

10. Wolf S, Zeisler C, Sint D, Romeis J, Traugott M, Collatz J. A simple and cost-effective molecular method to track predation on Drosophila suzukii in the field. J Pest Sci (2004). 2018 Mar 3;91(2):927–35.

11. Bourne A, Fountain MT, Wijnen H, Shaw B. Potential of the European earwig (Forficula auricularia) as a biocontrol agent of the soft and stone fruit pest Drosophila suzukii. Pest Manag Sci. 2019;75(12):3340–5.

12. Renkema JM, Telfer Z, Gariepy T, Hallett RH. Dalotia coriaria as a predator of Drosophila suzukii: Functional responses, reduced fruit infestation and molecular diagnostics. Biol Control. 2015;89:1–10.
