# Supplementary material for: DNA screening of Drosophila suzukii predators in berry field orchards shows new predatory taxonomical groups
Source: PLoS One. 2021 Apr 8;16(4):e0249673. doi: 10.1371/journal.pone.0249673 (PMC8031375; doi:10.1371/journal.pone.0249673)
Supplement: S2 Table — Spider families are grouped according to its functional group into Undefined/Specialists, web-building and hunting spiders. Spiders in studies where family was not specified were classified as Spiders. In the Type of study column, studies were considered as “Field” when the predators or potential predators were captured in the field or predation was observed in the field. Predation was considered “Positive” or “Negative” when specific predators predated or not on D. suzukii, respectively; it was considered “Not confirmed” when the study identified potential predators and predation was observed, but it was not possible to identify which specific arthropod was the predator. DNA presence was considered “Positive” if DNA amplification occurred with SWD specific primers, “Negative” when there was no DNA amplification with specific primers to identify SWD, and “Not tested” when predation was not assessed based on SWD DNA presence. Origin refers to the country where the experiments took place, either in the laboratory or field. (DOCX) [file pone.0249673.s003.docx]

**Table S2.** List of spiders considered as potential *D. suzukii* predators. Spider **families** are grouped according to its **functional group** into Undefined/Specialists, web-building and hunting spiders. Spiders in studies where family was not specified were classified as Spiders. In the **Type of study** column, studies were considered as “Field” when the predators or potential predators were captured in the field or predation was observed in the field. **Predation** was considered “Positive” or “Negative” when specific predators predated or not on *D. suzukii*, respectively; it was considered “Not confirmed” when the study identified potential predators and predation was observed, but it was not possible to identify which specific arthropod was the predator. **DNA presence** was considered “Positive” if DNA amplification occurred with SWD specific primers, “Negative” when there was no DNA amplification with specific primers to identify SWD, and “Not tested” when predation was not assessed based on SWD DNA presence. **Origin** refers to the country where the experiments took place, either in the laboratory or field.

| Group | Family | Type of study | Predation | DNA presence | | Origin | Reference |
| --- | --- | --- | --- | --- | --- | --- | --- |
| Undefined/Specialists | Eutichuridae, Linyphiidae, Mimetidae, Trechaledidae | Field | Negative | Negative | USA | | [1] |
|  | Immature spiders |  | Negative | Negative | USA  Switzerland | | [1,2] |
| Web-building | Theridiidae | Field | Positive | Positive | USA  Switzerland | | [1,2] |
|  | Agelenidae, Araneidae, Linyphiidae |  | Positive | Positive | Switzerland | | [2] |
|  | Agelenidae, Amaurobiidae, Araneidae, Cybaeidae, Dictynidae,  Hahnidae, Nephilidae, Pisauridae |  | Negative | Negative | USA | | [1] |
|  | Tetragnathidae |  | Negative | Negative | USA  Switzerland | | [1,2] |
| Hunting | Clubionidae, Salticidae | Field | Positive | Positive | USA  Switzerland | | [1,2] |
|  | Lycosidae, Philodromidae, Thomisidae |  | Negative | Negative | USA | | [1] |
|  | Oxyopidae |  | Positive | Positive | Switzerland | | [2] |
| Spiders | Not specified | Field | Positive | Not tested | USA | | [3] |
|  |  |  | Not confirmed | Not tested | USA | | [4,5] |

**References**

1. Schmidt JM, Whitehouse TS, Green K, Krehenwinkel H, Schmidt-Jeffris R, Sial AA. Local and landscape-scale heterogeneity shape spotted wing drosophila (Drosophila suzukii) activity and natural enemy abundance: Implications for trophic interactions. Agric Ecosyst Environ. 2019;272(November 2018):86–94.

2. Wolf S, Zeisler C, Sint D, Romeis J, Traugott M, Collatz J. A simple and cost-effective molecular method to track predation on Drosophila suzukii in the field. J Pest Sci (2004). 2018 Mar 3;91(2):927–35.

3. Woltz JM, Donahue KM, Bruck DJ, Lee JC. Efficacy of commercially available predators, nematodes and fungal entomopathogens for augmentative control of Drosophila suzukii. J Appl Entomol. 2015;139(10):759–70.

4. Kamiyama MT, Schreiner Z, Guédot C. Diversity and abundance of natural enemies of Drosophila suzukii in Wisconsin, USA fruit farms. BioControl. 2019;64(6):665–76.

5. Woltz JM, Lee JC. Pupation behavior and larval and pupal biocontrol of Drosophila suzukii in the field. Biol Control. 2017 Jul;110(April):62–9.
