# Supplementary material for: DNA screening of Drosophila suzukii predators in berry field orchards shows new predatory taxonomical groups
Source: PLoS One. 2021 Apr 8;16(4):e0249673. doi: 10.1371/journal.pone.0249673 (PMC8031375; doi:10.1371/journal.pone.0249673)
Supplement: S3 Table — (DOCX) [file pone.0249673.s004.docx]

**Table S3.** Spider families grouped in the corresponding functional group (identified according to Cardoso *et al.* [1]) and general group (web-building spiders, hunting spiders and other spiders).

| General Group | Specific Functional Group | Family |
| --- | --- | --- |
| Web-building spiders | Space web weaver | Dictynidae  Theridiidae |
|  | Orb web weaver | Araneidae  Uloboridae  Tetragnathidae |
|  | Sheet web weaver | Agelenidae |
| Hunting spiders | Ambush hunters | Thomisidae |
|  | Ground hunters | Lycosidae |
|  | Other hunters | Oxyopidae  Sparassidae  Cheiracanthiidae  Philodromidae  Salticidae |
| Other spiders | Specialists | Zodariidae |

**References**

1. Cardoso P, Pekár S, Jocqué R, Coddington JA. Global patterns of guild composition and functional diversity of spiders. PLoS One. 2011;6(6).
